# Macrophages and Cell-Cell Spread of HIV-1

**DOI:** 10.3390/v2081603

**Published:** 2010-08-05

**Authors:** Kayoko Waki, Eric O. Freed

**Affiliations:** Virus-Cell Interaction Section, HIV Drug Resistance Program, National Cancer Institute, Frederick, MD 21702, USA; E-Mail: wakik@mail.nih.gov

**Keywords:** HIV-1, macrophage, synapse, transmission

## Abstract

Macrophages have been postulated to play an important role in the pathogenesis of HIV-1 infection. Their ability to cross the blood-brain barrier and their resistance to virus-induced cytopathic effects allows them to serve as reservoirs for long-term infection. Thus, exploring the mechanisms of virus transmission from macrophages to target cells such as other macrophages or T lymphocytes is central to our understanding of HIV-1 pathogenesis and progression to AIDS, and is vital to the development of vaccines and novel antiretroviral therapies. This review provides an overview of the current understanding of cell-cell transmission in macrophages.

## 1. Introduction

Infection by HIV-1, a member of the genus *Lentiviridae*, results in an acute period characterized by high viral loads and a transient drop in CD4^+^ T cell counts. Following a vigorous adaptive immune response, viral loads decline and CD4^+^ T-cell counts recover. Over time, however, CD4 counts decline progressively, eventually leading to immunodeficiency and death [1]. HIV-1 enters target cells in most cases by using CD4 (a major attachment receptor), and chemokine coreceptors (CCR5 or CXCR4) [2]. CD4^+^ T cells represent a major target cell population *in vivo*. However, other cell populations, notably macrophages, are also productively infected by HIV-1. Unlike T cells, macrophages survive for long periods of time post-infection [3,4] and their ability to cross the blood-brain barrier suggests an important role in disseminating virus to the central nervous system [5].

## 2. Role of macrophages in lentiviral biology

Lentiviruses display the unique capacity among retroviruses to infect non-dividing cells. Macrophages are a terminally differentiated, non-dividing cell type that is central to lentiviral biology. The lentivirus genus includes, in addition to HIV-1, a number of viruses that infect horses (equine infectious anemia virus), sheep (maedi-visna virus), cattle (bovine immunodeficiency virus), goats (caprine arthritis-encephalomyelitis virus), cats (feline immunodeficiency virus), and non-human primates (simian immunodeficiency virus). These viruses all infect macrophages and in many cases cause neurological diseases [6]. FIV can cause immunodeficiency in domestic cats. SIVs are a diverse group of viruses that infect a wide variety of non-human primates. These viruses do not typically cause disease in African monkeys (their natural hosts), but in the Asian macaque (a non-natural host) they induce an AIDS-like illness [7]. Similar to HIV-1, SIV and FIV infect T cells as well as macrophages [8–11]. For SIV, SIV/HIV-1 chimeras (SHIVs), and FIV, tissue macrophages have been observed in some studies to be a major cell population producing infectious viruses in the affected organs, which include primary and secondary lymphoid tissues and the central nervous system [10,12]. Macrophages play important roles from the early stage through the latent period to the terminal symptomatic phase. Therefore, despite some notable differences with HIV-1-induced disease in humans, the macaque and feline models using SIV/SHIV and FIV, respectively, will be useful for understanding the biology of HIV-1 infection and disease development in humans and also the contribution of each cellular component, including macrophages [6].

## 3. HIV-1 replication

As mentioned above, HIV-1 targets immune cells – primarily T cells, macrophages, and dendritic cells – that express CD4 and chemokine coreceptors CCR5 or CXCR4. The levels of coreceptor expression differ between T-cell lines, primary CD4^+^ T cells, and macrophages. Most T-cell lines express high levels of CXCR4 but are CCR5-negative. In contrast, CCR5 is expressed abundantly on macrophages. Primary CD4^+^ T-cells generally express both CXCR4 and CCR5. Thus, CXCR4 (X4)-tropic strains of HIV-1 can infect T-cell lines, primary CD4^+^ T cells, but only to a limited extent macrophages; CCR5 (R5)-tropic strains infect monocyte-derived macrophages (MDMs) and primary CD4^+^ T cells; and dual-tropic (R5/X4) strains can infect all of these cell types. During the course of HIV-1 infection *in vivo*, a shift in coreceptor usage from R5 to R5/X4 and in some cases X4 often occurs [13]. This shift towards increased CXCR4 usage is typically accompanied by a decline in CD4^+^ cell numbers. It has been suggested that X4-tropic strains are more readily neutralized than R5-tropic isolates; the former thus emerge near the end of the disease course when the immune system has been largely incapacitated [14].

Because virus isolates present early in infection are typically R5-tropic, it has long been assumed that macrophages serve as an early target cell during person-to-person transmission. However, a recent study that used single genome sequencing to isolate and characterize transmitted (“founder”) viruses observed that these HIV-1 strains replicated poorly in macrophages despite being R5-tropic [15]. This finding, which extends earlier reports that CD4^+^ memory T-cells are the primary target for primate lentiviral infection early post-transmission [16], raises questions about the importance of macrophages in primary infection.

The HIV-1 replication cycle begins with the binding of the viral envelope (Env) glycoprotein complex – composed of the surface Env glycoprotein gp120 and the transmembrane Env glycoprotein gp41 – to CD4 and coreceptor on the target cell. These interactions induce conformational changes in gp120 and gp41 that lead to the exposure of the fusion peptide at the N-terminus of gp41. Fusion peptide insertion into the target membrane triggers the fusion of viral and host lipid bilayers and subsequent release of the viral core into the target cell cytoplasm [17]. After gaining access to the cytosol, the viral RNA genome is reverse transcribed to double-stranded DNA, which is translocated to the nucleus and integrated into the host cell genome. *In vitro* studies have shown that macrophages are capable of maintaining unintegrated viral DNAs for several weeks post-infection [18].

Once the HIV-1 genome has integrated into the host cell chromosome, it serves as the template for the synthesis of a number of viral RNAs, which are exported from the nucleus to the cytoplasm for translation and packaging. The Env glycoprotein precursor, gp160, is synthesized in the endoplasmic reticulum (ER) and transported to the plasma membrane via the secretory pathway. In contrast, the Gag and GagPol polyprotein precursors are synthesized on free ribosomes in the cytosol and are targeted to the plasma membrane by a poorly understood mechanism. Assembly generally takes place at the plasma membrane [19,20], and nascent particles bud off from the cell surface. So-called “late” domains near the C-terminus of Gag recruit components of the ESCRT (for endosomal sorting complex required for transport) machinery to promote the pinching-off of virus particles from the plasma membrane. This ESCRT machinery normally functions in membrane budding and fission reactions in late endosomes and during the abscission step of cytokinesis [21–24]. During or shortly after virus release, the viral protease (PR) cleaves the Gag and GagPol precursors, leading to virion maturation - a structural and morphological rearrangement of the virion that is essential for infectivity. With the completion of maturation, the mature virions can undergo a new round of infection.

## 4. HIV-1 Gag trafficking in macrophages

**Figure 1.**
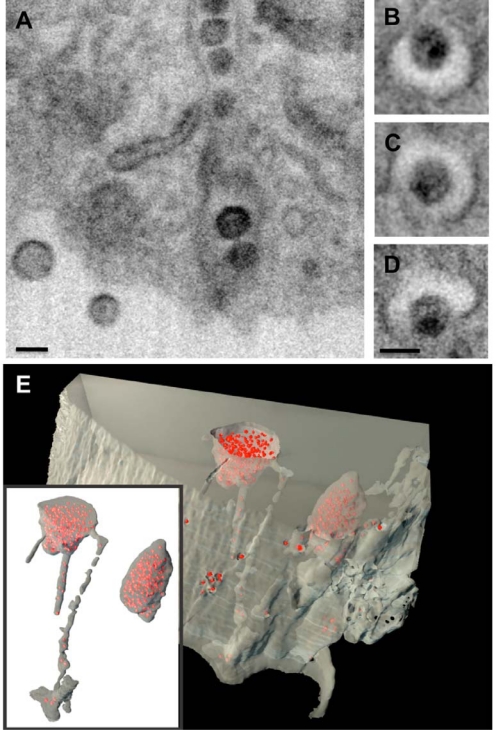
Assembly of HIV-1 in an internal compartment in primary monocyte-derived macrophages (MDMs). Ion-abrasion scanning EM shows the internal virus-containing compartments in MDMs and the virion channels connecting these compartments with the cell surface. **(A)** Transverse and **(B-D)** axial sections are shown, with scale bars = 100nm. **(E)** Reconstruction of a 3D image from IA-SEM, showing internal HIV-1 particles (red) and virion channels. Reprinted from Bennett *et al.* (2009) [32].

Although it is well established that the assembly and release of HIV-1 virions take place at the plasma membrane in T cells, the site of assembly in macrophages has been the subject of controversy [25]. Early electron microscopy (EM) data demonstrated that virus assembly in monocyte-derived macrophages in culture takes place in an internal, vesicle-like compartment [26]. This compartment was later shown to bear tetraspanin markers, such as CD63 and CD81, that are characteristic components of late endosomes or multivesicular bodies (MVBs) [27–29]. The prevailing hypothesis at that time was that in infected macrophages HIV-1 assembles in MVBs and is released from the cell upon fusion of the virus-containing MVBs with the plasma membrane – a pathway used to export exosomes from antigen-presenting cells [27–29]. More recently, Deneka *et al.* and Welsch *et al.* observed that this putative internal virus-containing compartment in macrophages is connected to the exterior of the cell by a thin channel, and is thus continuous with – and part of – the plasma membrane rather than being a late endosome or MVB [30,31]. Bennett and colleagues used a 3D imaging technique known as ion abrasion scanning EM to visualize the internal virus-containing compartment in MDMs [32]. Interestingly, many of the narrow channels connecting the internal virus-containing compartment with the cell surface contained numerous virions lined up “single file” [32] (Figure 1). An additional viewpoint was offered by Benaroch and colleagues, who observed HIV-1 assembly in MDMs in an intracellularly closed compartment that failed to acidify [33]. In their study, they observed that only 20% of the virus-containing compartments could be stained by a membrane-impermeable dye. Collectively, these studies suggest that the apparently intracellular compartment in which HIV-1 assembles in MDMs is distinct from conventional endosomes/MVBs. Consistent with this hypothesis, Gousset and colleagues showed that virions formed by a Gag mutant that assembles in MVBs failed to relocate to sites of cell-cell contact, in contrast to WT virions, which efficiently translocated to the synapse [34].

Numerous studies have observed colocalization of Gag and tetraspanin markers within infected MDMs and at macrophage-macrophage and macrophage-T cell contacts; however, what role these molecules play in HIV-1 replication remains an interesting and unresolved topic of research. Ruiz-Mateos *et al.* showed that knocking down CD63 expression in MDMs with siRNA affected neither particle production nor infectivity [35]. However, overexpression of tetraspanins or treatment of virus-producing cells with anti-tetraspanin antibodies was shown to disrupt particle production, reduce the infectivity of HIV-1 virions [36–38], and suppress virus-induced syncytium formation [39]. Furthermore, depletion of CD81 was reported to enhance virus transmission to target cells [37]. Opposing data on the role of endogenous CD63 were presented by Chen and colleagues who reported that CD63 depletion inhibited HIV-1 replication in MDMs [40]. Overall, these data suggest that tetraspanins play regulatory role(s) in particle infectivity and cell-cell virus transmission.

The Gag polyprotein precursor, Pr55^Gag^ – often referred to simply as “Gag” – is the viral protein predominantly responsible for driving HIV-1 assembly [19,20]. No other viral proteins are required for the generation of immature VLPs. Pr55^Gag^ is composed of four major domains arranged from N- to C-terminus: matrix (MA), capsid (CA), nucleocapsid (NC), and p6. Concomitant with virus budding and release, these domains are liberated from Pr55^Gag^ by PR-mediated cleavage. Each domain of Gag carries out distinct functions during the assembly process. MA bears several determinants – a covalently attached myristic acid and a highly basic patch of amino acid residues – that are critical for directing the membrane association of Gag. The basic patch also regulates the localization of virus assembly, as mutations in this region of MA induce a retargeting of virus assembly to MVBs [27]. CA promotes Gag multimerization during assembly and also forms the outer shell of the viral core during virion maturation. NC, by virtue of its RNA binding capability, also directs Gag-Gag multimerization and is responsible for packaging the viral RNA genome into virions. The p6 domain contains binding sites for the ESCRT machinery that, as mentioned above, is responsible for particle budding and release. Specifically, p6 binds the ESCRT-I component Tsg101 and the ESCRT-associated factor Alix [21,22,24,41].

As is the case with other steps in the HIV-1 replication cycle, Gag trafficking to the site of assembly requires the participation of host cell factors. In cells in which HIV-1 assembles at and buds from the plasma membrane, Gag is recruited to cholesterol/sphingolipid-enriched lipid raft domains at the plasma membrane [42]. This is reflected by raft association of Pr55^Gag^ [43–45] and also by the raft-like composition of the viral lipid bilayer [46–48]. Lipid rafts likely serve as a platform for Gag assembly and for incorporation of the Env glycoprotein complex into virions [49–51]. The lipid phosphoinositide, phosphatidylinositol-4,5-bisphosphate [PI(4,5)P_2_], also promotes the targeting of Gag to the inner leaflet of the plasma membrane. Depletion of PI(4,5)P_2_ redirects HIV-1 assembly to MVBs [52], and HIV-1 MA binds directly to PI(4,5)P_2_ [53–55]. HIV-1 MA shows specificity for PI(4,5)P_2_ and its interaction with this lipid triggers increased exposure of the covalently attached myristic acid moiety [54], thus stabilizing Gag interaction with the membrane.

As mentioned above, mutation of basic residues in MA or PI(4,5)P_2_ depletion causes HIV-1 assembly to be redirected to MVBs. It remains to be determined in these cases what signals in Gag are responsible for MVB targeting. One might reasonably assume that late domain motifs in p6, which are known to interact with ESCRT machinery, would play a major role. However, deletion of p6 does not affect the MVB localization of MA basic domain mutants [27]. While it has been suggested that HIV-1 Gag relocalizes ESCRT machinery away from the MVB to the plasma membrane [56], Welsch *et al.* showed that HIV-1 infection did not significantly affect the localization pattern of ESCRT and ESCRT-associated proteins [57]. This study suggests that ESCRT components are sufficiently abundant both at the plasma membrane and in MVBs to promote HIV-1 budding.

In addition to lipid rafts and PI(4,5)P_2_, a number of cellular proteins have been implicated in Gag trafficking. These include the clathrin adaptor complexes AP-1, AP-2, AP-3, [58–62], the Golgi-localized γ-ear containing, Arf-binding (GGA) proteins, the ADP ribosylation factors (Arfs) [63], SOCS1 [64], the kinesin KIF4 [65,66], the ubiquitin E3-ligase POSH [67] – which binds the ESCRT-associated protein Alix – and Annexin 2 [68]. The mechanisms by which these diverse factors influence Gag targeting remain to be elucidated. Proteomics analysis has revealed the presence of a large number of host proteins in HIV-1 particles produced from infected macrophages [69]; in most cases, the roles of these proteins in virus replication remains to be determined.

## 5. Virus spread at points of cell-cell contact: the immunological and virological synapse

HIV-1 can spread between cells either as cell-free virus or via cell-cell transfer. Early studies demonstrated that cell-cell spread is far more efficient than cell-free infection [70–72]; modes of virus transfer involving cell-cell contact are thus likely to play a major role in virus dissemination *in vivo*, particularly in crowded lymphoid tissue in which cell contacts are frequent. Cell-cell transfer also allows viral infections to spread in a manner less “visible” to the host immune system than cell-free transmission. Newly assembled virus particles could be stored internally until infected cells contact uninfected cells, leading to the organized recruitment of virus particles to the contact site.

**Figure 2.**
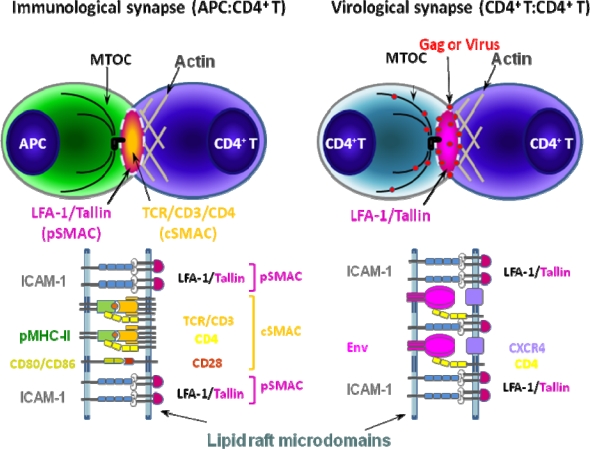
Schematic models of immunological synapse (IS) between APC and CD4^+^ T cell (left) and virological synapse (VS) between CD4^+^ T cells (right). The formation of an IS between APCs and CD4^+^ T cells begins with the recognition of antigenic peptide loaded on MHC-II (pMHC-II) on the APC by its cognate TCR/CD3/CD4 receptors on the CD4^+^ T cell. The adhesion molecules (ICAM-1 and LFA-1) and co-stimulatory molecules (CD80/86 and CD28) are also recruited to lipid raft microdomains (dotted line) at the IS to form the supramolecular activation complex (SMAC) [83]. These additional ligand-receptor interactions stabilize the complex. HIV-1-infected cells form a structurally similar contact, the VS, with uninfected cells. pSMAC, peripheral SMAC; cSMAC, central SMAC. Modified with permission from Piguet and Sattentau, 2004 [77].

It is now well established that HIV-1 and other retroviruses transfer from infected to uninfected cells at cell-cell contact sites that resemble immunological synapses (ISs) [73,74]. These have been termed virological synapses (VSs) [75–77] (Figure 2). ISs form between antigen-presenting cells (APCs) – such as macrophages and dendritic cells – and either CD4^+^ or CD8^+^ T cells. At the IS, antigenic peptide-loaded major histocompatibility complex (pMHC) on the APC and its cognate T-cell receptor (TCR)/CD3 complex on the T cell are recruited to the cell contact site. The pMHC-TCR interaction activates the T cell to induce its subsequent differentiation to become an effector cell. Additional ligand/receptor interactions stabilize the IS for prolonged periods of time to enable completion of the signaling event. The mediators of these interactions include intercellular adhesion molecule-1 (ICAM-1) on the APC, which binds to lymphocyte function-associated adhesion molecule (LFA-1) on the T cell; and CD28 on the T cell, which binds to CD80/CD86 on the APC. The recruitment of these molecules and the stability of the IS involves the actin/microtubule cytoskeletal network and lipid rafts [78–80]. Additional details on the IS and VS are reviewed in two recent papers [81,82].

The VS is formed between cells to transfer infectious viruses either newly assembled in the donor cell (*cis*-infection) or captured by the donor cell from the extracellular environment (*trans*-infection). The first HIV-1 cell-associated transfer across a synapse was demonstrated in the context of *trans*-infection from DCs to T lymphocytes [76], where viruses and their receptors were recruited to the contact sites. During the differentiation process, DCs appear to participate in different modes of transfer due to their differential susceptibility to HIV-1 infection [84]; immature DCs are relatively efficiently infected and can therefore transfer viruses by the *cis*-infection route. In contrast, direct infection of mature DCs is less efficient; these cells thus transfer virus primarily *in trans* [85]. Although *cis*- and *trans*-infection modes are different in terms of the origins of the virus being transferred, they share common features: 1) virus accumulates in tetraspanin-enriched compartments [62]; 2) HIV-1-harboring compartments are accessible to the cell surface [62,86]; and 3) the virus in the internal compartment is relocated to the VS upon contact with target cell [62]. Although the mechanism of virus recruitment to the VS in the *cis*-infection mode remains poorly studied, in *trans-*infection the recruitment of the following molecules has been observed: tetraspanins (CD81 and CD9) [85], DC-SIGN (dendritic cell-specific intercellular adhesion molecule-3-grabbing non-integrin), ICAM-1, and LFA-1 [87,88]. How the cytoskeletal network functions in DC-mediated *trans*-infection needs to be elucidated. However, recent studies indicate the involvement of the cytoskeletal network at multiple steps: the formation of the invaginated plasma-membrane-derived compartment is actin-dependent, but microtubule-independent [86]; actin-dependent macropinocytosis plays a role in the putative uptake of HIV-1 [89]; and actin and microtubules are involved in the formation of the synapse itself [89].

VS formation between T cells was first recognized in human T-cell leukemia virus type 1 (HTLV-1) infections [75]. Similar VS structures were also found in HIV-1-infected T cells [90], where viral molecules (Gag and Env), receptors, adhesion molecules, and actin were recruited to the VS. Cholesterol/GM1-enriched lipid rafts were shown to be required for VS formation and for the recruitment of Gag and Env [91]. Different cytoskeletal networks are involved on each side of the VS: F-actin on the target cells and both actin and tubulin on the infected cells [92]. Virus transfer and uptake seem to be carried out by an endocytic mechanism [93,94], probably in a clathrin- and dynamin-dependent manner [93]. Transfer can take place simultaneously from one infected cell to several uninfected cells at structures referred to as polysynapses [95]. A recent study observed that the VS is loosely structured and viral transfer across the VS can be inhibited by neutralizing antibodies [96]. To some extent, these observations challenge the view that the VS is a privileged microenvironment that is sequestered from the host humoral immune response.

Additional cell-associated viral transfer modes have been observed. These include the use of filopodial bridges, cytonemes, and tunneling nanotubes (TNT). These structures are actin-based, thread-like extensions of the plasma membrane, which can reach relatively distant target cells and require less contact area compared to synapses. Filopodial bridges have been shown to be utilized for the transfer of the retrovirus murine leukemia virus (MLV) in fibroblasts [97]. The MLV particles were observed to “surf” along the bridges, the formation of which required Env-receptor interactions. HIV-1 transfer was also found to move across filopodial bridges towards CD4/CXCR4-expressing cells [97].

TNTs, which allow the exchange of relatively large cytoplasmic components - including vesicles - between connected cells, are a well-established pathway for direct cell-to-cell communication [65]. HIV-1 has been reported to use TNTs for transfer between T cells and between macrophages [98,99]. Macrophages form TNTs of heterogeneous length, and increase in number during active viral replication [98]. Infected macrophages were observed to simultaneously form different types of TNTs in addition to filopodia [95].

## 6. Transfer of HIV-1 across the macrophage VS

**Figure 3.**
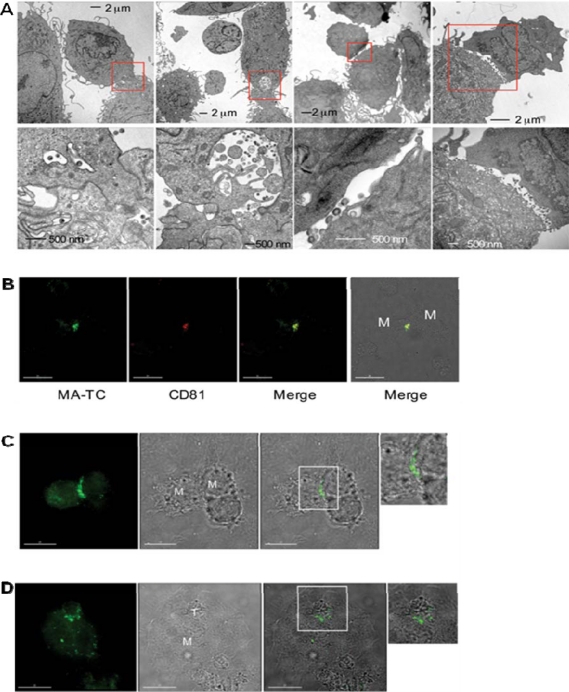
Recruitment and accumulation of Gag at the MDM/MDM and MDM/T-cell synapse. Cells were infected with a tetra-Cys-tagged HIV-1 clone **(A, B)** or an Env(-) derivative **(C, D)**. EM images show the accumulation of virions in intracellular compartments near the plasma membrane and the contact sites between infected MDM and uninfected MDM **(A)**. Gag and the tetraspanin CD81 are recruited to the MDM/MDM contact site **(B)**. Gag in infected MDMs is relocated to the contact sites with both uninfected MDMs **(C)** and Jurkat T cells **(D)** in the absence of Env expression. Scale bars, 30 μm **(B)** and 15 μm **(C)** and **(D)**. From Gousset *et al.* 2008 [34].

Compared to the T-cell and DC VS, relatively little is known about the formation of, and transfer of virus across, the VS in macrophages. Initial studies, however, suggest that much of what has been learned about the VS in T cells and DCs will apply to macrophages. Two recent studies showed that macrophages could form VSs to transfer HIV-1 to uninfected macrophages and T cells, with features similar to those seen in T cells and DCs [34,100] (Figure 3). Gousset and colleagues used the biarsenical probe FlAsH in living cells to study the localization and movement of HIV-1 Gag bearing a tetra-Cys tag – the binding site for FlAsH. The tetra-Cys tag, which was introduced near the C-terminus of the MA domain in Gag, was shown to not interfere with HIV-1 assembly and release or particle infectivity. In the infected MDMs, virus particles accumulated both at the plasma membrane and in internal compartments containing the tetraspanin markers CD81 and CD82 [34]. There appeared to be little movement of Gag between these two compartments during the time course of the live-cell imaging (∼90 min). However, upon coculture with T cells and subsequent VS formation, the internal virus moved rapidly (within 5–10 min) to the VS. In this system, Env was found not to be required for either VS formation or movement of Gag to the synapse [34], though in another study Env interactions with receptor and coreceptor were shown to be required for transfer of Gag across the synapse [100]. Interestingly, as mentioned above, a MA mutant that targets to MVBs also demonstrated localization to an internal tetraspanin-enriched compartment but did not translocate to the synapse [34]. This observation suggests that the WT and the mutant Gag assemble in different compartments in MDM; the most likely explanation for these findings is that the WT Gag assembles in the internal surface-connected compartment discussed above [30–32] rather than in true MVBs.

Groot and colleagues [100] cocultivated infected MDMs with autologous CD4^+^ T cells for up to six hours. Within one hour of coculture, Gag, Env, and CD4 could be observed accumulating at VSs. The VS formation appeared to be transient, with 5–10% of T cell becoming Gag-positive by six hours of coculture. The authors used a transwell system to show that whereas nearly 10% of T cells were virus-positive after 10 hours, when cell-cell contact was prevented only 0.5% of T cells became infected [100].

It remains unclear by what mechanism HIV-1 particles that assemble and accumulate in the internal virus-containing compartment traffic to the VS. This could occur by particle movement along the virion channel [32] to the cell surface or could result from the evagination of the internal compartment and wholesale “dumping” of particles at the VS. The live-cell imaging data of Gousset and colleagues [34] favor the latter hypothesis. Also remaining to be defined are the molecular signals that drive particle movement and/or evagination reactions. The recent studies discussed above [34,100] reported that T-cells often extend pseudopod or uropod-like extensions that contact the infected MDM at the VS; the signals that induce the formation of these T-cell extensions remain to be defined.

## 7. Perspectives

Macrophages have long been thought to play an important role in lentiviral biology, and it has been clear for decades that the site at which HIV-1 assembles in MDMs differs from the primarily plasma-membrane-associated assembly observed in T cells. However, many issues relating to HIV-1 infection of this cell type remain to be fully understood. For example, what precisely is the nature of the compartment in which virus assembles in MDMs? How are particles that assemble in the putative internal compartment exported to the exterior of the cell? What is the trigger for movement of internally assembled virions to the VS? What role do tetraspanins and other markers of the IS play in MDM synapse formation and movement of virus across the synapse? Also unclear is to what extent do observations made in MDM tissue culture systems apply to the *in vivo* biology of HIV-1. In any event, a more complete characterization of HIV-1 replication in this fascinating cell type will likely contribute to our understanding of HIV-1 biology and to the development of anti-HIV-1 vaccines and antiretroviral therapies.
